# Albumin and total bilirubin for severity and mortality in coronavirus disease 2019 patients

**DOI:** 10.1002/jcla.23412

**Published:** 2020-06-17

**Authors:** Ying Wang, Li Shi, Yadong Wang, Guangcai Duan, Haiyan Yang

**Affiliations:** ^1^ Department of Epidemiology School of Public Health Zhengzhou University Zhengzhou China; ^2^ Department of Toxicology Henan Center for Disease Control and Prevention Zhengzhou China

To the Editor,

The novel coronavirus disease 2019 (COVID‐19) has given rise to a worldwide pandemic. As of May 15, 2020, according to the latest data report from the World Health Organization, there have been 4 338 658 confirmed cases worldwide, resulting in 297 119 deaths.^1^ Previous papers have reported the abnormalities of albumin values and total bilirubin values among COVID‐19 patients.^2^ We aimed to investigate the differences in albumin values and total bilirubin values in the progression of COVID‐19 patients and to provide evidence for clinical treatment.

The databases of PubMed, Web of Science, and medRxiv were searched for related studies up to May 16, 2020. Search terms included the keywords: ([“coronavirus” or “COVID‐19” or “SARS‐CoV‐2” or “2019 nCoV”] and “laboratory”). There is no language restriction. Articles were screened by title, abstract, and full texts for the data. All articles that reported information on the albumin values or total bilirubin values in COVID‐19 patients with or without the severe disease (ie, ICU admission or acute respiratory distress syndrome) or between non‐survivors and survivors were included. The *I* ^2^ test was applied to assess the statistical heterogeneity among studies. The weighted mean difference (WMD) and 95% confidence interval (CI) were calculated to estimate the pooled effects of albumin values. The standardized mean difference (SMD) and 95% CI were calculated to estimate the pooled effects of total bilirubin values owing to different detection methods and data representation unit among studies.^3^ The sample size, median, and interquartile range (IQR) were used to estimate the mean and standard deviation.^4^ Egger's and Begg's tests were used to assess the publication bias. All statistical analyses were conducted using *Stata* 11 (StataCorp) software package, and *P* < .05 was considered significant.

Initially, a total of 2165 studies were identified in the search. By reading titles and abstracts, 2077 studies were excluded. By reading the full texts, 64 studies were excluded owing to that they did not report albumin values or total bilirubin values. At last, 24 studies were enrolled in our meta‐analysis.^5^, ^6^, ^7^, ^8^, ^9^, ^10^, ^11^, ^12^, ^13^, ^14^, ^15^, ^16^, ^17^, ^18^, ^19^, ^20^, ^21^, ^22^, ^23^, ^24^, ^25^, ^26^, ^27^, ^28^ The main characteristics of the included studies are shown in Table 1.

**Table 1:** Characteristics of the included studies

Author	Location	Age	Male	Severe/non‐survival			Non‐severe/survival		
				n	Albumin (g/L)	Total bilirubin	n	Albumin (g/L)	Total bilirubin
Peng Yudong et al^5^	China	62 (median)	53 (47.3)	16	NR	13.05 (mmol/L) (9.4‐16.65)	96	NR	11.4 (mmol/L) (8.7‐14.8)
Mo Pingzheng et al^6^	China	54 (median)	86 (55.5)	85	36 (32‐40)	NR	70	39 (36‐42)	NR
Huang Chaolin et al^7^	China	49 (median)	30 (73.2)	13	27.9 (26.3‐30.9)	14 (mmol/L) (11.9‐32.9)	28	34.7 (30.2‐36.5)	10.8 (mmol/L) (9.4‐12.3)
Qian Guoqing et al^8^	China	50 (median)	37 (40.7)	9	38.55 (36.33‐39.25)	NR	82	40.2 (38‐42.4)	NR
Wu Chaomin et al^9^	China	51 (median)	128 (63.7)	84	30.4 (27.15‐33.35)	12.9 (mg/dL) (9.5‐17.05)	117	33.7 (30.95‐36.3)	10.5 (mg/dL) (8.6‐13.65)
Liu Yanli et al^12^	China	55 (median)	59 (54.1)	53	NR	10.5 (mmol/L) (6.2‐14.8)	56	NR	8.3 (mmol/L) (6.8‐11.7)
Li Jie et al^11^	China	45.1 (mean)	9 (52.9)	12	NR	19.3 (mmol/L) ± 14.8	5	NR	12.2 (mmol/L) ± 4.8
Liu Jing et al^13^	China	48.7 (mean)	15 (37.5)	13	NR	13.2 (μmol/L) ± 5.5	27	NR	8.8 (μmol/L) ± 4.1
Liu Jingyuan et al^10^	China	40 (median)	31 (50.8)	17	43 (37‐45.5)	NR	44	44 (41‐47)	NR
Huang Huang et al^14^	China	44.87 (mean)	63 (50.4)	32	33.49 ± 4.79	NR	93	39.83 ± 4.49	NR
Liu Min et al^15^	China	35 (mean)	10 (33.3)	4	35 ± 1.1	NR	26	42 ± 3.4	NR
Liu Wei et al^16^	China	38 (median)	39 (50.0)	11	36.62 ± 6.6	NR	67	41.27 ± 4.55	NR
Ling Yun et al^17^	China	49.9 (mean)	154 (52.7)	21	35.8 ± 4.6	NR	271	40.9 ± 3.8	NR
Wang Dawei et al^28^	China	56 (median)	75 (54.3)	36	NR	11.5 (mmol/L) (9.6‐18.6)	102	NR	9.3 (mmol/L) (8.2‐12.8)
Wang Feng et al^18^	China	68.6 (mean)	21 (75.0)	14	NR	15.8 (μmol/L) ± 6.8	14	NR	9.1 (μmol/L) ± 3.6
Zheng F. et al^19^	China	45 (median)	80 (49.7)	30	NR	12.7 (μmol/L) (9.2‐16.9)	131	NR	10.7 (μmol/L) (8.18‐15.3)
Lei Shaoqing et al^20^	China	55 (median)	14 (41.2)	15	NR	13.0 (μmol/L) (9.1‐19.2)	19	NR	8.1 (μmol/L) (6.5‐12.9)
Yang Xiaobo et al^21^	China	59.7 (mean)	35 (67.0)	32	NR	19.5 (μmol/L) ± 11.6	20	NR	13.1 (μmol/L) ± 4.3
Zhou Fei et al^22^	China	56 (median)	119 (62.0)	54	29.1 (26.5‐31.3)	NR	137	33.6 (30.6‐36.4)	NR
Ruan Qiurong et al^23^	China	NR	102 (68.0)	68	28.8 ± 3.8	18.1 (μmol/L) ± 10.7	82	32.7 ± 3.8	12.8 (μmol/L) ± 6.8
Wang Yang et al^24^	China	64 (median)	179 (52.0)	133	31 (28‐34)	12.9 (mmol/L) (9.8‐19.2)	211	36 (33‐39)	8.5 (mmol/L) (6.3‐11.3)
Wu Chaomin et al ^9^	China	58.5 (median)	60 (71.4)	44	29.1 (26.2‐31.55)	14.5 (mg/dL) (10.35‐19.8)	40	31.35 (27.7‐34.2)	11.65 (mg/dL) (9.33‐15.15)
Tomlins Jennifer et al^25^	US	75 (median)	60 (63.0)	20	30 (24‐32)	NR	75	32 (27‐36)	NR
Wang Dawei et al^26^	China	51 (median)	57 (53.3)	19	NR	11.3 (mmol/L) (9.4‐20.7)	88	NR	9.5 (mmol/L) (8.4‐12.9)
Wang Kun et al^27^	China	NR	140 (47.3)	19	NR	10.2 (μmol/L) (5.9‐17)	277	NR	8.2 (μmol/L) (5.5‐11.8)

Note

All values are n (%), median (IQR), or mean ± SD.

Abbreviation: NR, not reported.

A total of 2948 COVID‐19 patients, totaling in 24 studies, reported albumin values or total bilirubin values, of these, 1713 patients in 17 studies were divided into severe and non‐severe groups, and 1319 patients in 8 studies were divided into non‐survival and survival groups. The pooled WMD indicated that the albumin values were significantly lower in COVID‐19 patients with the severe disease than in those without by using a random‐effects model (WMD = −4.39 g/L, 95% CI: −5.64, −3.15; *P* < .001; *I* ^2^ = 70.2%, *P* = .001) (Figure 1A). The result of SMD showed that total bilirubin values were considerably higher in COVID‐19 patients with the severe disease than in those without by using a fixed‐effects model (SMD = 0.51, 95% CI: 0.36, 0.66; *P* < .001; *I* ^2^ = 10.9%, *P* = .343) (Figure 1B). The changes of albumin levels and total bilirubin levels in non‐survival patients versus survival patients were similar to that in severe patients versus non‐severe patients by using a random‐effects model (Albumin WMD = −4.06 g/L, 95% CI: −4.98, −3.14; *P* < .001; *I* ^2^ = 49.2%, *P* = .096. Total bilirubin SMD = 0.71, 95% CI: 0.50, 0.91; *P* < .001; *I* ^2^ = 38.6%, *P* = .149) (Figure 1C,D). No publication bias was found by Egger's test (all *P* > .05) and Begg's test (all *P* > .05).

**FIGURE 1:**
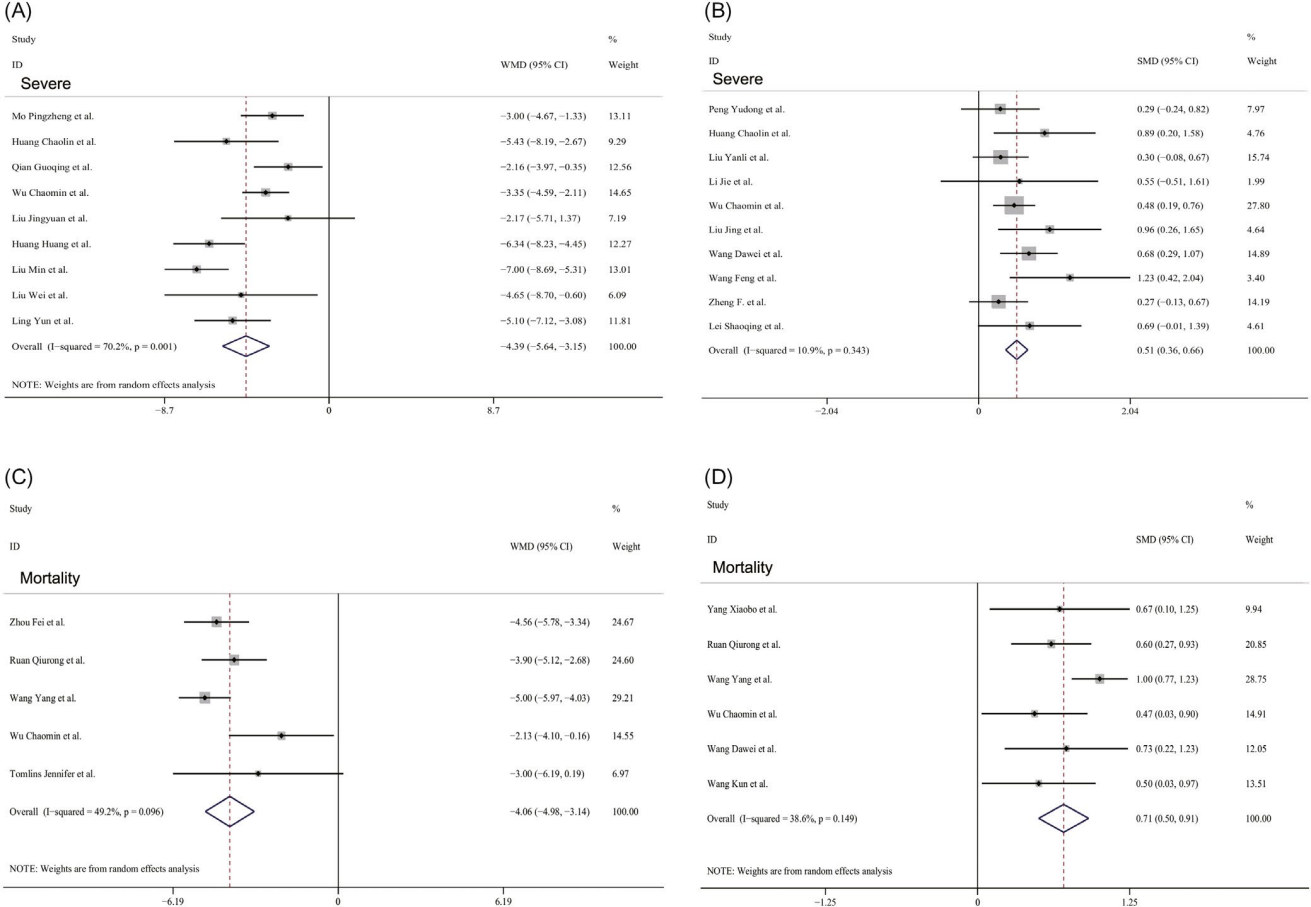
Forest plots of albumin values (A) and total bilirubin values (B) in severity patients with COVID‐19, and forest plots of albumin values (C) and total bilirubin values (D) in mortality patients with COVID‐19

Our results demonstrated that there were significant differences in albumin values and total bilirubin values in COVID‐19 patients with varying degrees of disease severity, suggesting that alterations in these two biomarkers’ values can predict changes in the patients’ condition. Besides, studies have shown that patients with COVID‐19 have elevated alanine aminotransferase and aspartate aminotransferase values,^29^ suggesting that patients with COVID‐19 may have liver damage. Our results also supported this notion that COVID‐19 infection could cause liver damage in other aspects to a certain extent. However, the majority of patients in the included studies came from China, and the sample size was limited. Further analyses including more studies are needed to verify our findings.

## Funding information

This work was supported by a grant from the National Natural Science Foundation of China (grant number 81973105). The funder has no role in the preparation of manuscript and decision to submission.

## References

[1] World Health Organization, Coronavirus disease 2019 (COVID‐19) . https://www.who.int/docs/default‐source/coronaviruse/situation‐reports/20200515‐covid‐19‐sitrep‐116.pdf?sfvrsn=8dd60956_2. Accessed May 15, 2020.

[2] Huang Y , Tu M , Wang S , et al. Clinical characteristics of laboratory confirmed positive cases of SARS‐CoV‐2 infection in Wuhan, China: a retrospective single center analysis. Travel Med Infect Dis. 2020:101606. 10.1016/j.tmaid.2020.101606 [Epub ahead of print]. 32114074 PMC7102650

[3] Deeks JJ , Higgins JPT . Analysing data and undertaking meta‐analyses. In: Deeks JJ , Higgins J , Altman DG , eds. Cochrane Handbook for Systematic Reviews of Interventions, 2nd edn. Chichester, UK: John Wiley & Sons; 2019:241‐284.

[4] Wan X , Wang W , Liu J , Tong T . Estimating the sample mean and standard deviation from the sample size, median, range and/or interquartile range. BMC Med Res Methodol. 2014;14:135. 25524443 10.1186/1471-2288-14-135 PMC4383202

[5] Peng YD , Meng K , Guan HQ , et al. Clinical characteristics and outcomes of 112 cardiovascular disease patients infected by 2019‐nCoV. Zhonghua xin xue guan bing za zhi. 2020;48:E004. 10.3760/cma.j.cn112148-20200220-00105 32120458

[6] Mo P , Xing Y , Xiao Y , et al. Clinical characteristics of refractory COVID‐19 pneumonia in Wuhan, China. Clin Infect Dis. 2020:ciaa270. 10.1093/cid/ciaa270 [Epub ahead of print].

[7] Huang C , Wang Y , Li X , et al. Clinical features of patients infected with 2019 novel coronavirus in Wuhan, China. Lancet (London, England). 2020;395(10223):497‐506. 10.1016/S0140-6736(20)30183-5 PMC7159299 31986264

[8] Qian GQ , Yang NB , Ding F , et al. Epidemiologic and Clinical Characteristics of 91 Hospitalized Patients with COVID‐19 in Zhejiang, China: a retrospective, multi‐centre case series. QJM. 2020:hcaa089. 10.1093/qjmed/hcaa089 [Epub ahead of print]. PMC7184349 32181807

[9] Wu C , Chen X , Cai Y , et al. Risk factors associated with acute respiratory distress syndrome and death in patients with Coronavirus Disease 2019 Pneumonia in Wuhan, China. JAMA Intern Med. 2020:e200994. 10.1001/jamainternmed.2020.0994 [Epub ahead of print]. PMC7070509 32167524

[10] Liu J , Liu Y , Xiang P , et al. Neutrophil‐to‐lymphocyte ratio predicts severe illness patients with 2019 novel coronavirus in the Early Stage; 2020. 10.1186/s12967-020-02374-0 PMC7237880 32434518

[11] Li J , Li S , Cai Y , et al. Epidemiological and clinical characteristics of 17 hospitalized patients with 2019 novel coronavirus infections outside Wuhan, China; 2019.

[12] Liu Y , Sun W , Li J , et al. Clinical characteristics and progression of 2019 novel coronavirus‐infected patients concurrent acute respiratory distress syndrome; 2020.

[13] Liu J , Li S , Liu J , et al. Longitudinal characteristics of lymphocyte responses and cytokine profiles in the peripheral blood of SARS‐CoV‐2 infected patients. EBioMedicine. 2020;55:102763. 32361250 10.1016/j.ebiom.2020.102763 PMC7165294

[14] Huang H , Cai S , Li Y , et al. Prognostic factors for COVID‐19 pneumonia progression to severe symptom based on the earlier clinical features: a retrospective analysis; 2020. 10.3389/fmed.2020.557453 PMC7571455 33123541

[15] Liu M , He P , Liu HG , et al. Clinical characteristics of 30 medical workers infected with new coronavirus pneumonia. Zhonghua jie He He Hu Xi Za Zhi. 2020;43(3):209‐214. 32164090 10.3760/cma.j.issn.1001-0939.2020.03.014

[16] Liu W , Tao ZW , Wang L , et al. Analysis of factors associated with disease outcomes in hospitalized patients with 2019 novel coronavirus disease. Chin Med J. 2020;133(9):1032‐1038. 32118640 10.1097/CM9.0000000000000775 PMC7147279

[17] Ling Y , Lin Y , Qian Z , et al. Clinical analysis of risk factors for severe patients with novel coronavirus pneumonia. Chin J Infect Dis. 2020;38. 10.3760/cma.j.cn311365-20200211-00055 [Epub ahead of print].

[18] Wang F , Yang Y , Dong K , et al. Clinical characteristics of 28 patients with diabetes and COVID‐19 in Wuhan, China. Endocrine Pract. 2020:10.4158/EP‐2020‐0108. 10.4158/EP-2020-0108 [Epub ahead of print]. PMC7414317 32357072

[19] Zheng F , Tang W , Li H , et al. Clinical characteristics of 161 cases of corona virus disease 2019 (COVID‐19) in Changsha. Eur Rev Med Pharmacol Sci. 2020;24(6):3404‐3410. 32271459 10.26355/eurrev_202003_20711

[20] Lei S , Jiang F , Su W , et al. Clinical characteristics and outcomes of patients undergoing surgeries during the incubation period of COVID‐19 infection. EClinicalMedicine. 2020;21:100331. 10.1016/j.eclinm.2020.100331 [Epub ahead of print]. 32292899 PMC7128617

[21] Yang X , Yu Y , Xu J , et al. Clinical course and outcomes of critically ill patients with SARS‐CoV‐2 pneumonia in Wuhan, China: a single‐centered, retrospective, observational study. The Lancet Respiratory medicine. 2020;8(5):475‐481. 32105632 10.1016/S2213-2600(20)30079-5 PMC7102538

[22] Zhou F , Yu T , Du R , et al. Clinical course and risk factors for mortality of adult inpatients with COVID‐19 in Wuhan, China: a retrospective cohort study. Lancet (London, England). 2020;395(10229):1054‐1062. 10.1016/S0140-6736(20)30566-3 PMC7270627 32171076

[23] Ruan Q , Yang K , Wang W , Jiang L , Song J . Clinical predictors of mortality due to COVID‐19 based on an analysis of data of 150 patients from Wuhan, China. Intensive Care Med. 2020;46:846‐848. 32125452 10.1007/s00134-020-05991-x PMC7080116

[24] Wang Y , Lu X , Chen H , et al. Clinical Course and Outcomes of 344 Intensive Care Patients with COVID‐19. Am J Respir Crit Care Med. 2020;201(11):1430‐1434. 32267160 10.1164/rccm.202003-0736LE PMC7258632

[25] Tomlins J , Hamilton F , Gunning S , et al. Clinical features of 95 sequential hospitalised patients with novel coronavirus 2019 disease (COVID‐19), the first UK cohort. J Infect. 2020:S0163‐4453(20)30232‐2. 10.1016/j.jinf.2020.04.020 [Epub ahead of print]. PMC7184992 32353384

[26] Wang D , Yin Y , Hu C , et al. Clinical course and outcome of 107 patients infected with the novel coronavirus, SARS‐CoV‐2, discharged from two hospitals in Wuhan, China. Critical Care (London, England). 2020;24(1):188. 10.1186/s13054-020-02895-6 PMC7192564 32354360

[27] Wang K , Zuo P , Liu Y , et al. Clinical and laboratory predictors of in‐hospital mortality in patients with COVID‐19: a cohort study in Wuhan, China. Clin Infect Dis. 2020:ciaa538. 10.1093/cid/ciaa538 [Epub ahead of print]. PMC7197616 32361723

[28] Wang D , Hu B , Hu C , et al. Clinical Characteristics of 138 Hospitalized Patients With 2019 Novel Coronavirus‐Infected Pneumonia in Wuhan, China. JAMA. 2020;323(11):1061‐1069. 32031570 10.1001/jama.2020.1585 PMC7042881

[29] Rodriguez‐Morales AJ , Cardona‐Ospina JA , Gutiérrez‐Ocampo E , et al. Clinical, laboratory and imaging features of COVID‐19: A systematic review and meta‐analysis. Travel Med Infect Dis. 2020;34:101623. 32179124 10.1016/j.tmaid.2020.101623 PMC7102608

